# Proteus syndrome with sciatic nerve fibrolipomatous hamartoma: an uncommon finding in a rare disease: report of two cases with literature review

**DOI:** 10.1259/bjrcr.20210153

**Published:** 2021-12-13

**Authors:** Dalia Ibrahim

**Affiliations:** 1 Department of Radiology, Kasr Al Ainy Hospital, Cairo, Egypt

## Abstract

Proteus syndrome is an extremely rare condition, characterized by progressive asymmetric overgrowth of multiple body tissues. Here, we present two cases of Proteus syndrome demonstrating typical clinical and radiological features of Proteus syndrome, in addition to an uncommon fibrolipomatous hamartoma of the sciatic nerve. The first case is a 5-year-old girl who presented with seizures. The patient showed facial dysmorphic features, left head enlargement, kyphoscoliosis, asymmetric overgrowth of the right lower limb, right foot drop, and cribriform connective tissue nevi on the right palm and the right sole. Radiological examinations demonstrated left calvarial hyperostosis, dysplasia of the left cerebral hemisphere, dysregulation of the subcutaneous adipose fat of the body, kyphoscoliosis, and lipoma of the filum terminale. CT of both thighs showed asymmetric soft tissue overgrowth of the right thigh, associated with diffuse enlargement and fatty infiltration of the right sciatic nerve starting from the upper thigh, down to its bifurcation into the tibial and common peroneal nerves. The second case is an 18-year-old girl who presented with left conductive deafness. The patient showed facial dysmorphic features, right head enlargement, asymmetric overgrowth of the right upper limb, kyphoscoliosis, left foot drop, and cribriform connective tissue nevi on the nose and the left foot. Radiological examinations demonstrated right calvarial hyperostosis, left external auditory canal hyperostosis and stenosis, and kyphoscoliosis. CT and MRI of both thighs showed diffuse enlargement of the left sciatic nerve starting from the upper thigh down to the mid-thigh and showing interfascicular adipose tissue proliferation, giving the typical features of nerve lipomatosis.

## Case presentation

### Case 1

**Figure 1.**
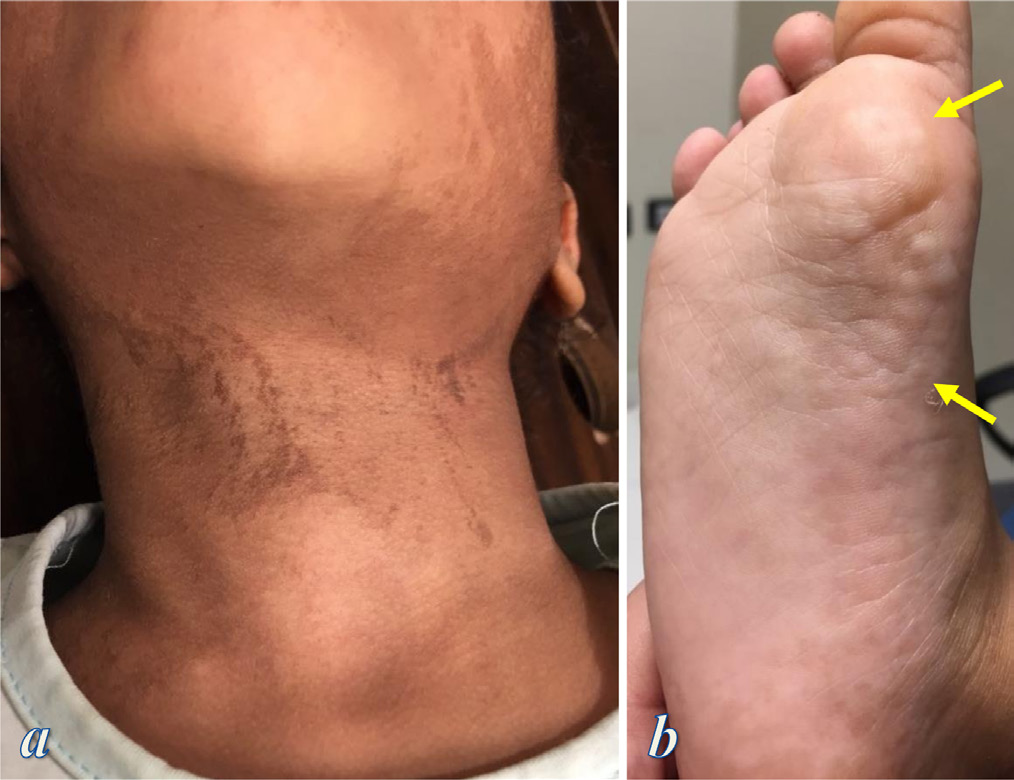
Clinical photographs of the first patient; (**a**) photograph showing liner epidermal nevi on the patient’s neck and the left side of the face, (**b**) photograph of the right sole shows cribriform connective tissue nevi (arrows)

A 5-year-old girl originated from the second pregnancy of non-consanguineous healthy parents. She was delivered vaginally at 37 weeks of gestation. Upon birth, she presented normal weight, length, and morphology, apart from a small left head bump which was thought to be related to birth trauma. At 2 months of age, she started to develop linear epidermal nevi on her face and neck. During infancy, her mother noticed delayed mental and motor milestones, as well as progressive asymmetric enlargement of the left head bump as well as the right lower limb. Later, the patient started to develop seizures, strabismus, and multiple subcutaneous soft swellings on the torso. Physical examination of the patient showed left head enlargement, facial dysmorphic features in the form of long face, bilateral low lying palpebral fissures, strabismus, depressed nasal bridge, wide anteverted nares, open mouth at rest, multiple dental caries, and linear epidermal nevi on the face and the neck (Figure 1a). The patient’s torso showed kyphoscoliosis and multiple subcutaneous soft (lipoma-like) swellings on the front and back of her body. She also had asymmetrically enlarged right lower limb showing mild venous varicosities. Cribriform connective tissue nevi were seen on the right palm and the right sole (Figure 1b). Neurological examination showed severe mental retardation, abnormal gait, and right foot drop. The patient had no previous diagnosis for her congenital deformities and there was no history of similar illness in the family.

The patient met all general clinical criteria of Proteus syndrome which are sporadic occurrence, mosaic distribution, and progressive course. She also developed most of the specific criteria of Proteus syndrome; she had cribriform connective tissue nevi on the palm and sole (Criterion A), linear epidermal nevi on the face and neck (Criterion B1), skull, vertebral and right lower limb asymmetrical overgrowth (Criterion B2), adipose tissue dysregulation (Criterion C1), venous vascular malformations (Criterion C2), facial phenotype of Proteus syndrome (Criterion C3).

### Investigations

**Figure 2.**
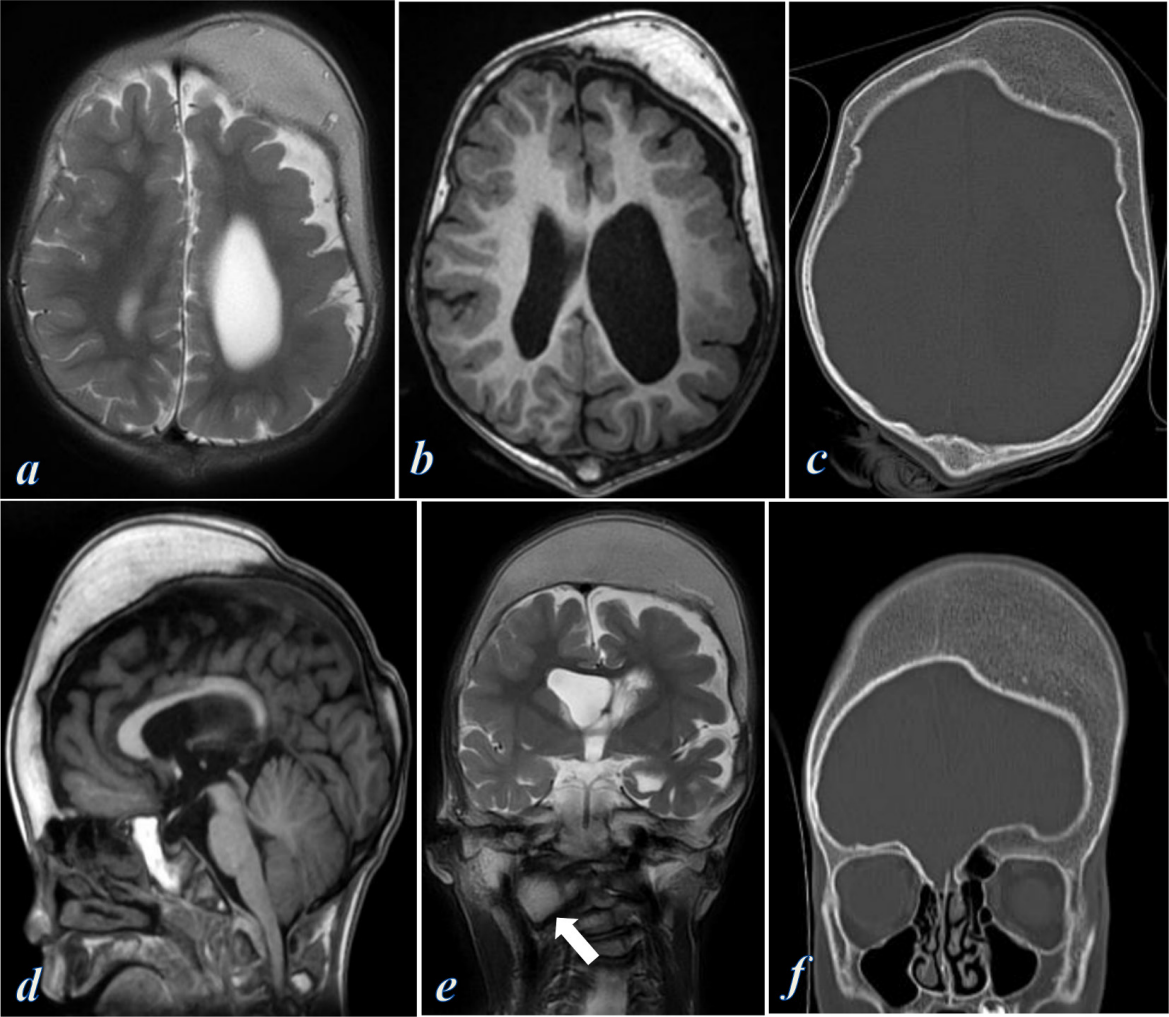
MRI and CT examination of the brain; (**a**) axial T_2_ weighted image shows asymmetric left frontoparietal calvarial thickening eliciting high signal, with dysplastic changes of the left cerebral hemisphere showing thick cortex, cortical pachygyria, abnormal grey–white matter differentiation, thin white matter, and large left lateral ventricle. (**b**) Axial *T*_1_ weighted image shows left frontoparietal calvarial thickening eliciting fatty marrow signal. (**c**) CT axial bone-window images showed asymmetrical thickening (hyperostosis) of the left frontal and occipital bone. (**d**) Sagittal *T*_1_ weighted image shows calvarial thickening of fatty marrow signal involving the frontal, parietal, sphenoid and occipital bones, with soft tissue hypertrophy of the atlantoaxial ligaments causing narrowing of the foramen magnum. (**e**) Coronal *T*_2_ weighted image shows significant left calvarial thickening, left cerebral atrophy and deep periventricular gliosis, and abnormal overgrowth of the right lateral mass of C1 vertebra (arrow) with subsequent cervical scoliosis. (**f**) CT coronal bone-window images showed asymmetrical calvarial thickening (hyperostosis) of the left frontal, maxillary, temporal and sphenoid bones.

CT and MRI of the brain showed asymmetric calvarial thickening (hyperostosis) of the left frontal, parietal, sphenoid, maxillary, temporal, and occipital bones, which elicit fatty marrow signal on MRI images (Figure 2). The left cerebral hemisphere underlying the left calvarial thickening appeared dysplastic showing cortical pachygyria, abnormal grey–white matter differentiation, thin white matter, enlarged left lateral ventricle (Figure 2a and b). Additionally, there was asymmetrical soft tissue hypertrophy of the atlantoaxial ligaments on the right side causing foramen magnum narrowing (Figure 2d), as well as asymmetrical overgrowth of the right side of C1 vertebra with subsequent cervical scoliosis (Figure 2e).

**Figure 3.**
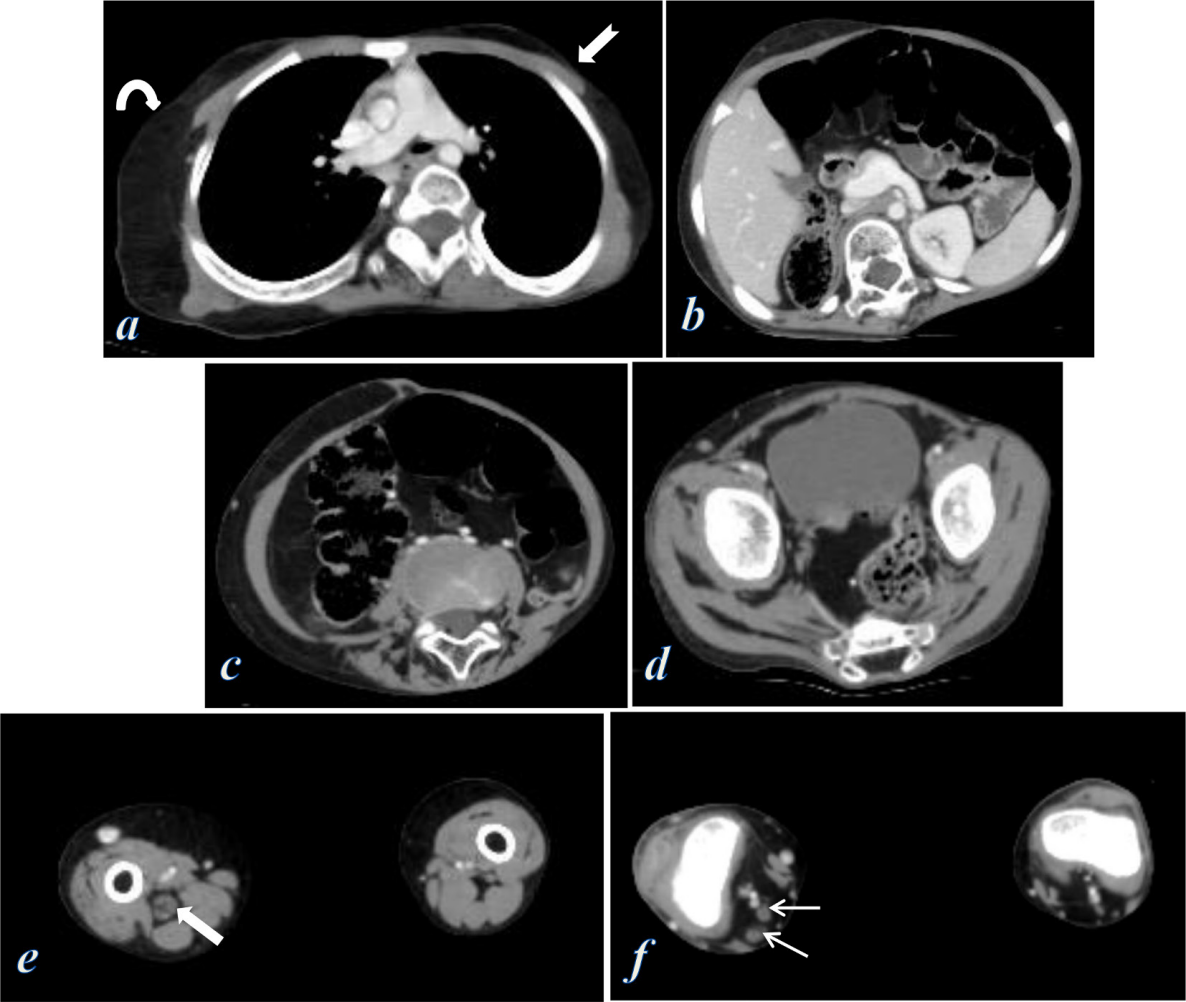
Contrast-enhanced CT scan of the chest, abdomen, pelvis and thighs. (**a**) Axial image of the chest shows asymmetric subcutaneous adipose fat of the chest wall with areas of increased fat deposition (curved arrow) and other areas of absent subcutaneous fat (notched arrow). (**b**) Axial image of the upper abdomen shows venous ectasia of the portal, splenic and superior mesenteric veins. (**c, d**) Axial images of the lower abdomen show irregular asymmetric intra-abdominal and pelvic lipomatosis (evident on the right paracolic gutter and right pararectal region), and irregular asymmetric subcutaneous fat deposition of the abdominal wall with fatty infiltration of the gluteal and erector spinae muscles, also note the venous ectasia of the superficial veins of the abdominal wall. (**e, f**) Axial images of both thighs show asymmetrically enlarged right thigh with muscular and subcutaneous fat hypertrophy, venous ectasia of the great and short saphenous veins, and diffuse enlargement of the right sciatic nerve (thick arrow), tibial and peroneal nerves (thin arrows) showing interfascicular fat density with no post-contrast enhancement.

Contrast-enhanced CT scan of the chest, abdomen, pelvis, and both thighs demonstrated dysregulation of the subcutaneous adipose fat of the chest and abdominal wall with regions of subcutaneous fat hypertrophy alternating with regions of subcutaneous fat atrophy (Figure 3a–d), associated with fatty infiltration of the erector spinae and gluteal muscles (Figure 3c and d). Intra-abdominal and pelvic asymmetric peritoneal lipomatosis, evident along the right paracolic gutter and the right para-rectal region (Figure 3c and d). The patient also showed multiple venous vascular malformations in the form of venous ectasia of the portal and superior mesenteric veins (Figure 3b), as well as the superficial veins of the abdominal wall, and the saphenous veins of the right lower limb (Figure 3c–f).

CT of both thighs demonstrated asymmetrically enlarged right thigh with muscular and subcutaneous fat hypertrophy, associated with diffuse enlargement of the right sciatic nerve starting from the upper thigh down to its bifurcation into the tibial and common peroneal nerves and showing interfascicular fat density (Figure 3e and f). Unfortunately, the patient was lost before MRI of the thighs was made.

**Figure 4.**
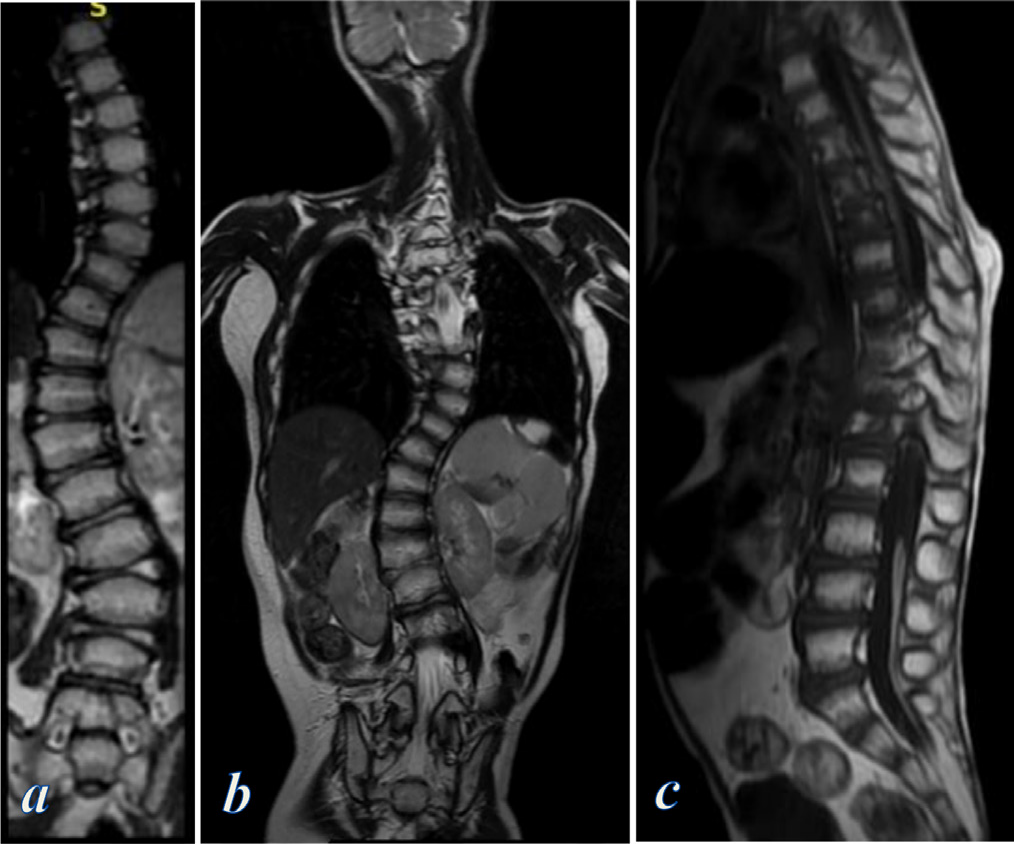
MRI examination of the spine. (**a**) Coronal *T*_2_ weighted image shows S-shaped scoliotic deformity of the dorsolumbar spine secondary to asymmetrical vertebral overgrowth. (**b**) Coronal *T*_2_ weighted image shows vertebral scoliosis and irregular subcutaneous fat deposition of the chest and abdominal wall. (c) Sagittal *T*_1_ weighted image shows kyphotic deformity of the dorsal spine with a focal increase of the subcutaneous fat of the mid-back. Lipoma of the filum terminale is seen opposite L3 down to S3

MRI of the dorsolumbar spine showed kyphoscoliosis secondary to asymmetrical overgrowth of the right side of D11 and L1 vertebral bodies (Figure 4). Lipoma of the filum terminale was also noted (Figure 4c).

### Case 2

**Figure 5.**
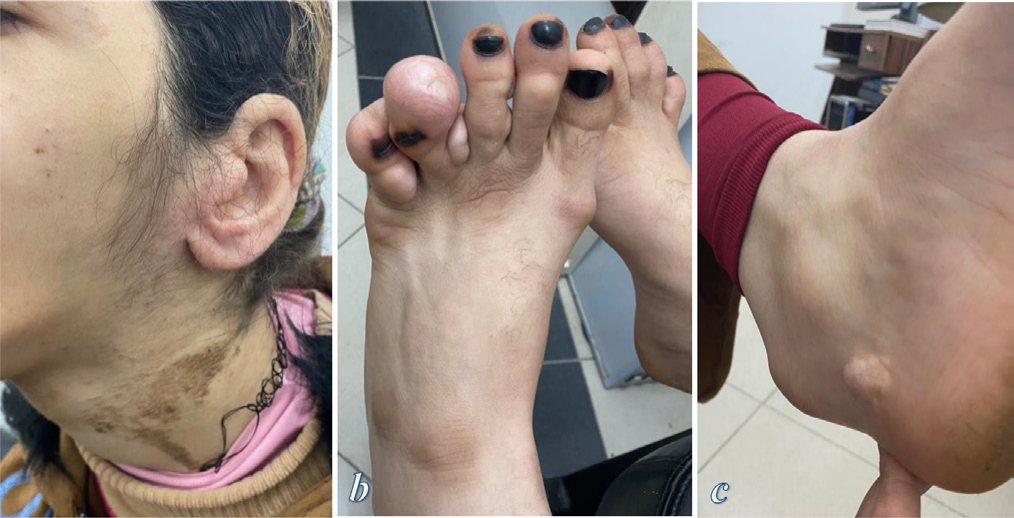
Clinical photographs of the second patient. (**a**) Photograph showing liner epidermal nevi on the patient’s neck. (**b, c**) Photograph of the left foot shows cribriform connective tissue nevi on the third, fourth and fifth toes and a smaller connective tissue nevus on the posteromedial of the left foot (**c**).

An 18-year-old girl originated from the first pregnancy of non-consanguineous healthy parents. Upon birth, she presented normal weight, length, and morphology. During infancy, she had delayed mental and motor milestones. She developed cribriform connective tissue nevus on her nose that was surgically removed, but it later recurred. The patient later developed connective tissue nevi on the left foot and multiple linear epidermal nevi on her neck. That was followed by asymmetric enlargement of the right side of the head and face as well as the right upper limb. The patient suffered from left conductive hearing loss. Physical examination of the patient showed tall stature, facial deformity with asymmetrically enlarged right side of the head and face and cribriform connective tissue nevus on her nose, as well as linear epidermal nevi on her neck (Figure 5a). The patient’s torso showed kyphoscoliosis, an asymmetrically enlarged right upper limb. Her left foot showed cribriform connective tissue nevi on the third, fourth and fifth toes and along the posteromedial aspect of the foot (Figure 5b and c). Neurological examination showed moderate mental retardation, abnormal gait, and left foot drop. The patient had no previous diagnosis for her congenital deformities and there was no history of similar illness in the family.

The patient met all general clinical criteria of Proteus syndrome which are sporadic occurrence, mosaic distribution, and progressive course. She also showed many of the specific criteria of Proteus syndrome; she had cribriform connective tissue nevi on the nose and foot (Criterion A), linear epidermal nevi on the neck (Criterion B1), skull, vertebral and right upper limb asymmetrical overgrowth (Criterion B2).

## Investigations

**Figure 6.**
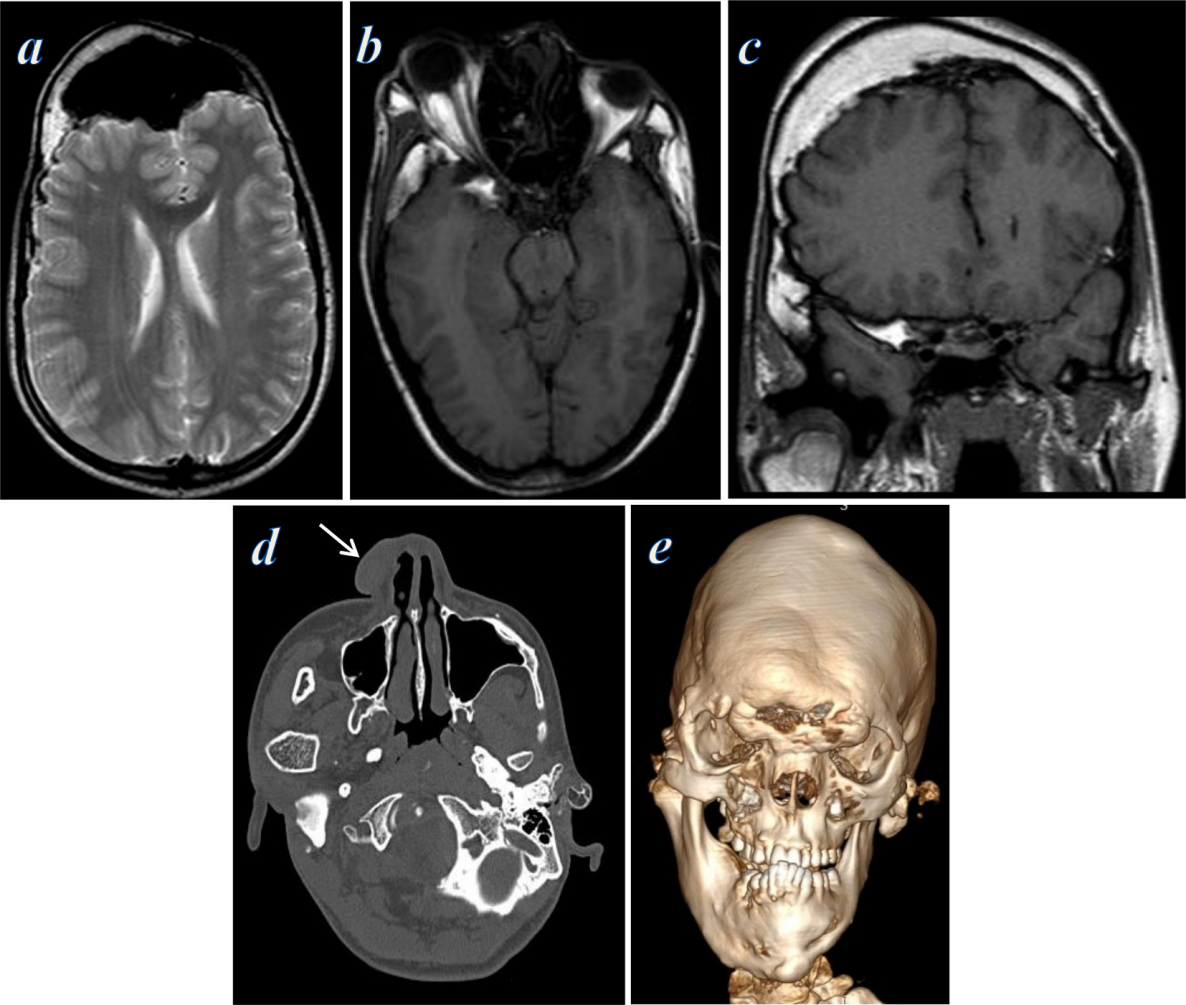
MRI and CT examination of the brain and face. (**a**) Axial *T*_2_ weighted image shows asymmetric right frontal calvarial thickening and hyperpneumatized frontal sinus. Normal MRI of the brain. (**b**) Axial *T*_1_ weighted image shows right sphenoid and temporal calvarial thickening and right eye proptosis. (**c**) Coronal *T*_1_ weighted image shows right frontoparietal, temporal and sphenoid calvarial thickening of fatty marrow signal, and abnormal overgrowth of the right mandibular condyle, sclerosis of the mandibular fossa of the right temporal bone, a sequel of prior infection and surgical intervention. (**d**) CT axial bone-window images showed asymmetrical overgrowth of the right mandibular condyles, left external auditory canal hyperostosis and stenosis, and right nasal cribriform connective tissue nevus (arrow). (**e**) 3D volume rendering bone window revealed facial deformity with asymmetrical overgrowth and hyperostosis of the frontal bone and the right side of the mandible. 3D, three-dimensional.

CT and MRI of the brain showed asymmetric calvarial thickening (hyperostosis) of the right frontal, temporal, and sphenoid bones, hyperpneumatized right frontal sinus, and right eye proptosis (Figure 6). Also, there was an asymmetrical overgrowth of the right mandibular condyle and ramus, nasal cribriform connective tissue nevus, and left external auditory canal hyperostosis and stenosis.

**Figure 7.**
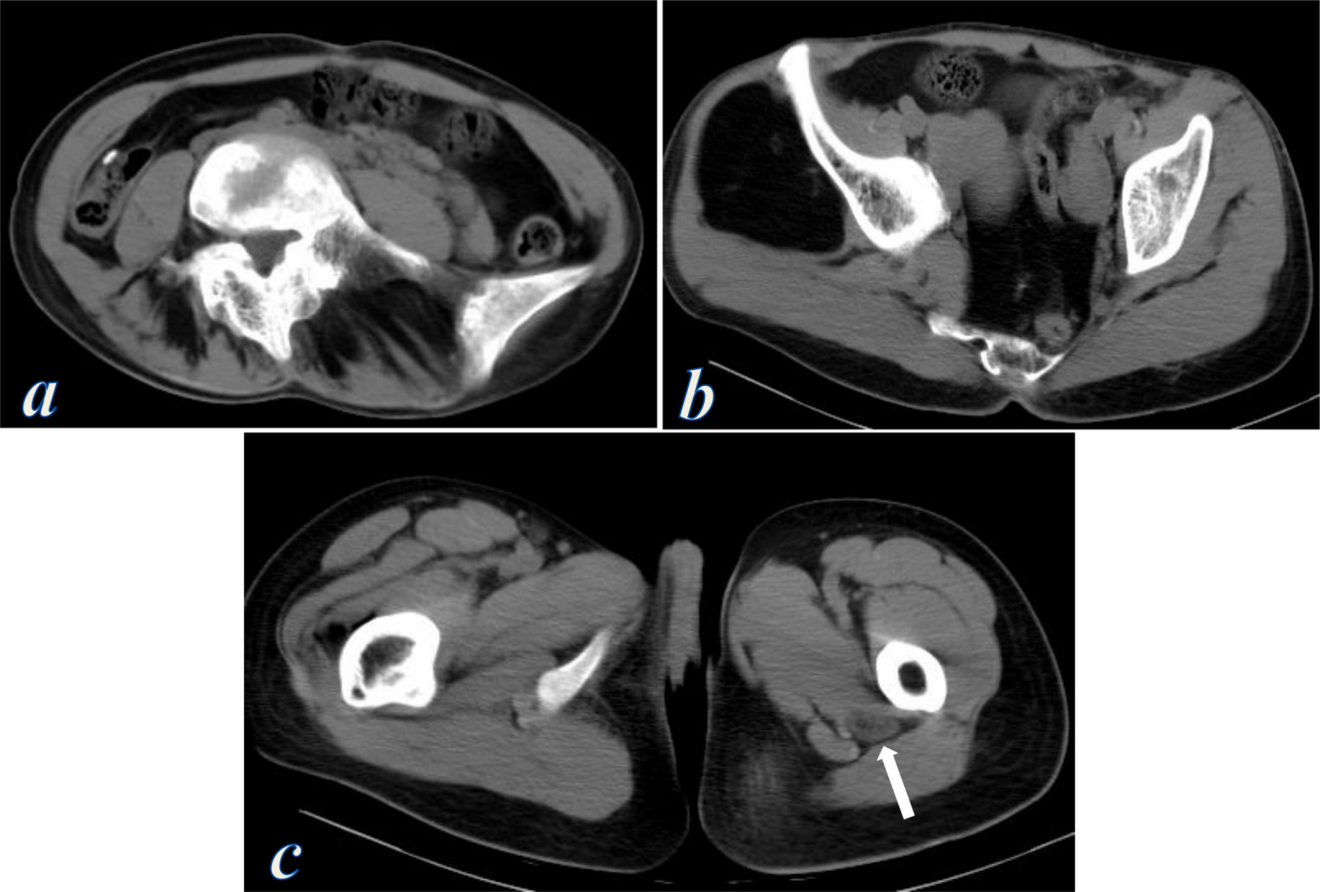
CT scan of the abdomen, pelvis, and thighs. (**a**) Axial image of the abdomen shows fatty infiltration of the erector spinae muscles. (**b**) Axial images of the pelvis show asymmetric intra-abdominal and pelvic lipomatosis (evident on the right paracolic gutter and pararectal regions) and a large right gluteal intramuscular lipoma. (**c**) Axial image of both thighs shows diffuse enlargement of the left sciatic nerve showing fatty infiltration (arrow).

CT scan of the abdomen and pelvis shows fatty infiltration of the erector spinae muscles, a large right gluteal intramuscular lipoma, and intra-abdominal and pelvic lipomatosis along the right paracolic gutter and pararectal regions. CT scan of both thighs shows a diffuse enlargement of the left sciatic nerve extending from the upper thigh to the middle thigh and showing fatty infiltration (Figure 7).

**Figure 8.**
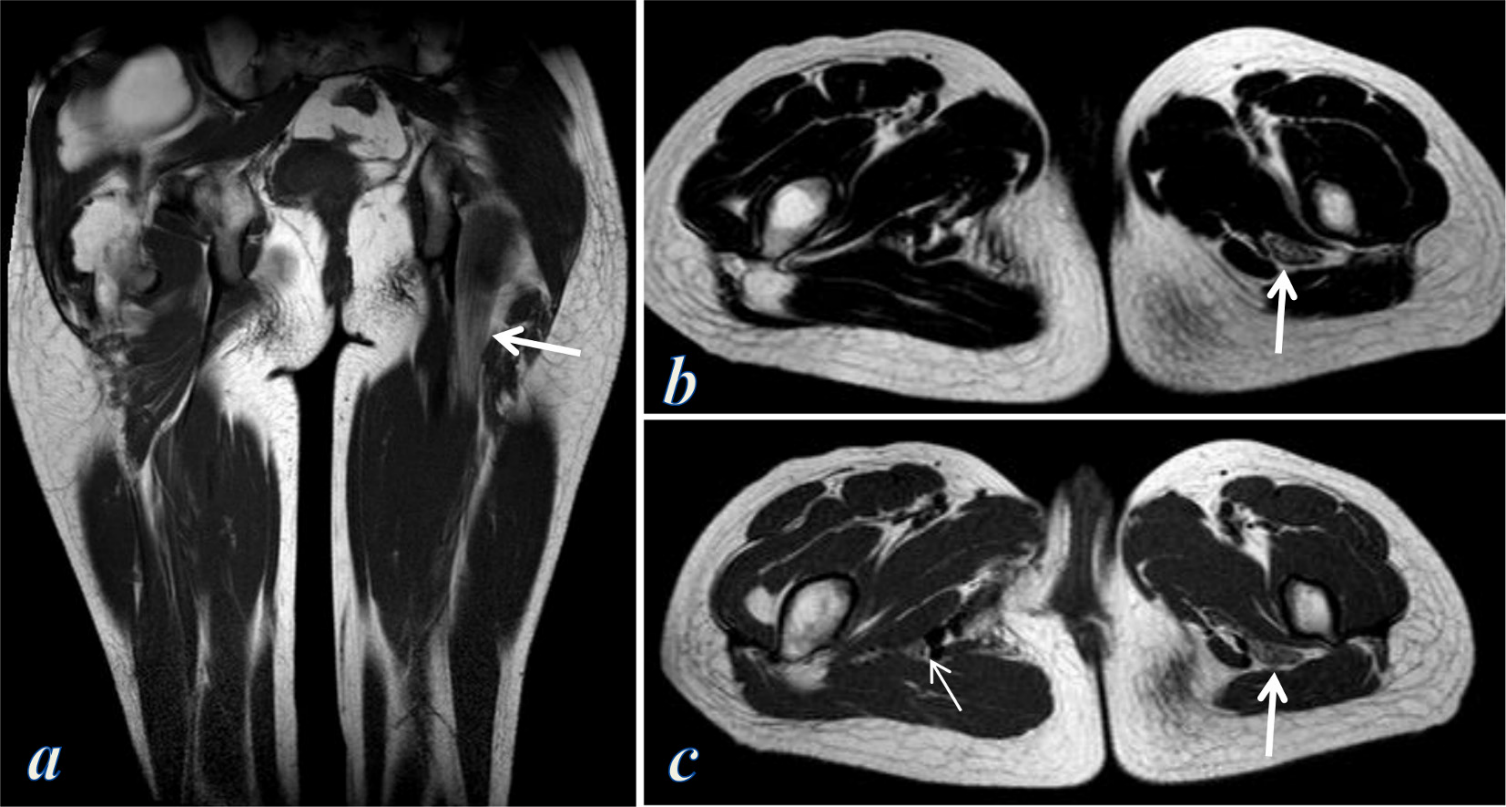
MRI examination of the thighs. (**a**) Coronal *T*_2_ weighted image shows diffuse enlargement of the left sciatic nerve with interfascicular fat proliferation eliciting high signal, giving the spaghetti appearance (arrow), right gluteal intramuscular lipoma is also noted. (**b, c**) Axial *T*_2_- and *T*_1_ weighted images show diffuse enlargement of the left sciatic nerve with interfascicular fat proliferation eliciting high signal, giving the coaxial cable appearance (thick arrows), compare to the normal contralateral right sciatic nerve (thin arrow in c).

MRI of both thighs showed diffuse enlargement of the left sciatic nerve starting from the upper thigh down to the mid-thigh. It shows interfascicular adipose tissue proliferation, giving the coaxial cable appearance on axial images and the spaghetti appearance on coronal images, compatible with nerve lipomatosis (Figure 8).

## Treatment

Both patients were managed by a multidisciplinary team consisting of specialists in neurology for seizures, orthopedics for kyphoscoliosis and limb asymmetry, plastic surgery for the skull hyperostosis, ENT for the hearing loss, dermatologists for the connective tissue nevi, and neurosurgeons for the sciatic nerve fibrolipomatous hamartomas. The first patient left the hospital before she continues the treatment plan. The second patient refused surgical treatment for the sciatic nerve hamartoma and instead got medical and physiotherapeutic treatment.

## Discussion

Proteus syndrome is a complex disorder characterized by progressive asymmetric tissue overgrowth. The disease was first described by Cohen and Hayden in 1979 as a new hamartomatous syndrome.^1^ It was further delineated by Wiedemann et al in 1983 who proposed the name Proteus after the Greek god of the sea who could change his shape to elude capture, as an impression of the significant variability of the patient’s shape during growth time.^2^ The first case was described in the 19th century by Sir Treves who described Joseph Merrick, the so-called “Elephant male” who was originally thought to have neurofibromatosis, yet recently it is believed he actually suffered from Proteus syndrome.^3^

Proteus syndrome is an extremely rare disease with an incidence of less than one per million live births.^4^ The disease is caused by a somatic activating mutation in AKT1 gene which is not acquired from a parent, yet it emerges randomly in one cell during embryological life. This gene is responsible for the regulation of cell growth, therefore mutation in this gene will disrupt the regulation of normal growth rendering it grow and divide normally. As cells proceed to grow and divide, some cells will have the mutation and others will not. This is known as mosaicism.^5^

Patients usually have few or no signs of the disease at birth. Signs of the disease start to appear between 6 and 18 months of age, get more severe during childhood, and tend to plateau after adolescence.^6^ The pattern of overgrowth can affect almost any part of the body. It mostly affects the skeleton, skin, adipose, and central nervous system.^7^

### Craniofacial manifestations

Individuals with Proteus syndrome may present clinically with seizures and/or mental retardation.^8^ The skull may exhibit asymmetric bony overgrowth (hyperostosis) of fatty marrow signal. Brain abnormalities include hemimegalencephaly, migrational disorders, polymicrogyria, pachygyria, dilated ventricles, hypoplastic white matter, abnormal grey–white matter differentiation, and thickened leptomeninges.^8,9^ Brain anomalies are usually located ipsilateral to the cranial hyperostosis.^10^ Facial manifestations include facial asymmetry secondary to asymmetrical bony or soft tissue overgrowth^9^ and facial phenotype which is usually associated with mental deficits or brain malformations. Facial phenotype manifested by a long face, down slanting palpebral fissures, a low nasal bridge with wide nostrils, and open mouth at rest.^9^ Several ocular abnormalities may be present such as strabismus, enlarged eye globe, epibulbar cysts, and dermoids.^11^

### Chest manifestations

Patients may demonstrate hamartomatous pulmonary cystic changes, lung consolidations, or problems attributed to skeletal abnormalities (*e.g.* scoliosis), or pulmonary embolism secondary to lower limb deep venous thrombosis which is the commonest cause of death.^12^

### Abdominal manifestations

Abdominal manifestations include visceromegaly of certain organs such as the spleen, kidneys, or thymus gland.^13^

### Musckolskeletal manifestations

#### Spine

Kyphoscoliosis is an important criterion as a result of asymmetrical vertebral body growth.^13^

#### Extremities

Asymmetric hemihypertrophy of the limbs, partial gigantism, and macrodactyly are very common findings.^14^

#### Soft tissues

Soft tissue abnormalities include asymmetrical subcutaneous fat distribution (dysregulation of the adipose fat) which usually occurs in the torso and also in the extremities.^9^

#### Skin

Skin manifestations are very common and sometimes pathognomonic in Proteus syndrome. Cribriform connective tissue nevus is very specific and sufficient for the diagnosis of Proteus syndrome when present, it’s commonly seen in the sole of the foot, palms of hands, or less likely chest, abdomen, and nose, and is characterized by deep gyrations similar to the brain surface.^9,15^ Linear epidermal nevus appears early in life and is usually located in the lateral zones of the trunk, back, or extremities.^12^ Café-au-lait patches may also be observed.^12^

#### Vascular manifestations

Vascular malformations are very common and include capillary, venous, or lymphatic types. Vascular ectasia and venous stasis may be progressive and could be complicated by deep venous thrombosis and subsequent pulmonary embolism.^16^

For doctors to consider a diagnosis of Proteus syndrome, the patient should have all three of the general characteristics in addition to some specific characteristics. The specific characteristics are grouped into three categories: A, B, and C. Diagnosis of Proteus syndrome requires all three general features to be present and either one feature from Category A, or two features from Category B, or three features from Category C (Table 1).^17,18^

**Table 1.** Diagnostic criteria of Proteus syndrome

**General criteria** Sporadic occurrence Mosaic distribution Progressive course
**Specific criteria**
**Category A** Cribriform connective tissue nevus
**Category B** Linear epidermal nevus. Asymmetric, disproportionate overgrowth (at least one of the following): Limbs Hyperostosis of the skull Hyperostosis of the external auditory canal Megaspondylodysplasia Viscera: spleen/thymus Specific tumors before being 20 years old of age Bilateral ovarian cystadenoma Parotid monomorphic adenoma
**Category C** Abnormal growth and/or distribution of fat (dysregulated fatty tissue overgrowth) (either of the following): Fatty tumors (lipomas) Lack of subcutaneous fat (regional lipohypoplasia) Vascular malformations (including one of the following): Capillary malformation Venous malformation Lymphatic malformation Lung bullae Facial features (all of the following): A long and narrow head (dolichocephaly) Long face Down slanting palpebral fissures and/or minor dropping of the eyelids (ptosis) Depressed nasal bridge Wide anteverted nares Open mouth at rest.

Lipomatosis of the nerve, previously known as lipofibromatous hamartoma, hamartoma of the nerve, or fibrolipomatous hamartoma, is a very rare tumor-like condition of the nerve with specific pathological and radiological characteristics.^19^ It is characterized by fusiform enlargement of the nerve secondary to fatty and fibrous tissue proliferation mainly in the epi- and perineurium that surrounds and infiltrates the nerves; hence it’s considered a hamartoma rather than a true tumor.^20^ The etiology is not yet clear. Some theories believe it is congenital, whereas, others consider it acquired, provoked by factors such as nerve irritation or trauma. It’s now thought that the true etiology might be a combination of both factors.^20^

Nerve lipomatosis commonly occurs in the median nerve and have been associated with macrodactyly occurring in the distribution of the affected nerve demonstrating osseous overgrowth and proliferation of the subcutaneous fat, a condition known as macrodystrophia lipomatosa (some authors consider it forme fruste/localized form of Proteus syndrome).^21^ Furthermore, nerve lipomatosis has been reported to be associated with overgrowth syndromes such as Klippel–Trenaunay–Weber syndrome (KTWS)^22,23^; however, the association between lipomatosis of the nerve and Proteus syndrome is not well known. To the best of our knowledge, there are only a few reports of Proteus syndrome with nerve lipomatosis^24–26^; a 5-year-old girl diagnosed with Proteus syndrome presented with hand pain, surgical exploration and pathology revealed fibrolipomatous hamartoma of the median nerve.^24^ Another case was a 4-year-old boy with features of Proteus syndrome presented with hand paraesthesias, surgical exploration revealed median nerve enlargement impressive of nerve lipomatosis.^25^The third case was a 13-year-old boy with a history of Proteus syndrome with left lower limb overgrowth. MRI revealed sciatic nerve fibrolipomatous hamartoma on the same side of the overgrown limb.^26^

Fibrolipomatous hamartoma of the sciatic nerve is additionally extremely rare and have been reported a few times in the literature^27–30^; neither of those cases were associated with Proteus syndrome.

MRI features of fibrolipomatous hamartoma are pathognomonic and are usually obviating the need for biopsy for diagnosis.^21^ There is a characteristic fusiform enlargement of the nerve, caused by fatty proliferation and thickening of nerve bundles. Nerve bundles appear as serpentine tubular structures, hypointense on both *T*_1_- and *T*_2_ weighted images, surrounded by fatty proliferation of high *T*_1_ signal and low signal in fat-suppressed images, giving the characteristic coaxial cable appearance on axial images and spaghetti appearance on coronal images.^27^

Radiological examinations are important tools for the diagnosis of Proteus syndrome and differentiation of this syndrome from similar hamartomatous diseases. It clearly shows the internal organ involvement and the hidden features of the disease.^31^ In our case, different radiological studies aided to distinguish different body swellings, such as the calvarial hyperostosis which was misdiagnosed as fibrous dysplasia, and the dysregulation of the adipose fat which was misdiagnosed as lipomas. CT and MRI of the brain helped to assess underlying brain abnormalities such as cerebral hemiatrophy and cortical pachygyria. CT also helped to identify the hidden venous vascular malformations involving the splanchnic veins, and the fibrolipomatous hamartomas involving the deep nerves. Recognition of the typical imaging findings of the disease can lead to an early diagnosis of this condition.^32^

The main differentials for this condition include other hamartomatous and overgrowth syndromes such as^13^;

- Neurofibromatosis Type 1 is usually distinguished from Proteus syndrome clinically by the presence of the clinical stigmata of neurofibromatosis namely cafe´-au-lait spots, Lisch nodules, and axillary freckling.^33^
- KTWS, in which symptoms are usually present at birth in contrast to Proteus syndrome in which the changes are usually absent at birth. Bone involvement and progressive hypertrophy is characteristic of Proteus syndrome and is usually absent in KTWS.^34^
- Congenital lipomatous overgrowth, vascular malformations, and epidermal nevi syndrome or hemihyperplasia and multiple lipomatosis syndrome; in both syndromes, symptoms are present at birth and are usually severe. The disease isn’t progressive; additionally, some criteria such as connective tissue nevi and skull hyperostosis are not present in those syndromes.^34^

**Treatment** of Proteus syndrome is multidisciplinary, including clinical, surgical, orthopedic, dermatological, and psychological support. The most life-threatening complications include deep vein thrombosis and pulmonary embolism, which may have a late diagnosis due to their very low incidence in pediatric patients.^35^ Treatment options for nerve lipomatosis include conservative approaches such as simple decompression, decompression with debulking of epineural tissue, external or internal neurolysis or excision of the hamartoma with or without nerve grafting.^24^ However, the role of those procedures for sciatic nerve lipomatosis is still unknown.^28^

## Learning points

- Proteus syndrome is an extremely rare disorder characterized by progressive asymmetric tissue overgrowth.
- The pattern of overgrowth can affect almost any part of the body. It mostly affects the skeleton, skin, adipose, and central nervous system.
- The disease has typical clinical and radiological features characteristic for this disease.
- Cribriform connective tissue nevi, epidermal nevi, skull hyperostosis, scoliosis, dysregulation of the subcutaneous fat, limb asymmetry, and vascular malformations are among important features of the disease.
- Nerve lipomatosis is a very rare tumor-like condition of the nerve with specific pathological and radiological characteristics. It commonly involves the median nerve, and rarely occurs in the sciatic nerve.
- Nerve lipomatosis have been associated with macrodactyly, a condition known as macrodystrophia lipomatosa and with overgrowth syndromes such as KTWS; however, its association with Proteus syndrome is not well known.
- Radiological examinations are important tools for identification of the hidden features of the disease and differentiation of this syndrome from similar hamartomatous diseases.
